# Improving case detection of tuberculosis among children in Bangladesh: lessons learned through an implementation research

**DOI:** 10.1186/s12889-017-4062-9

**Published:** 2017-01-28

**Authors:** Ziaul Islam, Kazi Istiaque Sanin, Tahmeed Ahmed

**Affiliations:** 1 Health Systems and Population Studies Division, International Centre for Diarrhoeal Disease Research, Bangladesh (icddr,b), Dhaka, 1212 Bangladesh; 2 Nutrition and Clinical Services Division, International Centre for Diarrhoeal Disease Research, Bangladesh (icddr,b), Dhaka, 1212 Bangladesh

**Keywords:** Tuberculosis, Child TB, Bangladesh, Implementation research, Child TB awareness

## Abstract

**Background:**

According to the Bangladesh National Tuberculosis Control Program (NTP), the proportion of childhood tuberculosis (TB) among all reported cases is only 3%. This is considerably lower compared to other high-burden countries. One of our previous studies identified substantial gaps at the primary care level related to capacity of service providers, supply of required logistics and community awareness about childhood TB. Therefore, we conducted an implementation study with the objectives to address those gaps.

**Methods:**

This implementation research was designed with pre and post-test evaluation at selected primary care facilities in urban and rural areas. Three interventions were implemented: (1) Training on childhood TB management for all categories of service providers (2) mass awareness campaign among primary and secondary school students and their teachers, mothers of <5y children, religious and community leaders and (3) facilitation of logistics supply at the study facilities. Training was conducted following the national guideline. We developed posters, leaflets, flipcharts and organized folksongs and street dramas as awareness campaign strategy. Quarterly follow up meetings were held with the facility managers of the study clinics. Cross-sectional surveys were conducted at the baseline and end line alongside review of service statistics to compare the change in community awareness and case detection of childhood TB.

**Results:**

Awareness regarding childhood TB among all target audience increased significantly showing better understanding of child TB symptoms, transmission, duration and treatment option. Overall proportion of TB case detection among children increased in all three sites compared to baseline as well as NTP estimate with relatively higher proportion in urban site. Majority of the children were suffering from extra-pulmonary TB and there were more female TB cases than male. However, supply and maintenance of necessary diagnostics and child friendly TB drugs remained suboptimal.

**Conclusion:**

Through implementation research, detection of childhood TB cases increased in all study facilities exceeding the NTP’s estimate. Community awareness on childhood TB improved significantly across all study sites as well. The NTP should implement strategies to raise community awareness alongside increasing the capacity of service providers and ensuring availability of diagnostics and pediatric TB drugs at the primary care level.

## Background

The magnitude of childhood tuberculosis burden has remained as uncharted territory for the global health policy makers. The World Health Organization’s (WHO) estimates in 2015 showed that 1 million children under 15 years of age suffer from tuberculosis (TB) worldwide, and more than 136,000 HIV negative children die each year from TB [1]. Many experts believe that these numbers grossly underestimate the true extent of the problem [2, 3] and probably much greater given the challenge in diagnosing childhood TB and reporting [4]. In high burden TB settings it has been estimated that 15–20% of all TB cases are among children, whereas in low burden TB settings it is around 2–7% [5]. However, among the notified new TB cases in south-east Asia and Sub-Saharan Africa, less than 4% are among children; likely due to poor case detection that is a result of multifaceted problems like low quality sample collection, pauci-bacillary nature of the disease in young children and lack of mycobacterial culture facilities in settings where TB and malnutrition are endemic [6]. The infected and diseased children, if not appropriately identified and treated, create a pool from which as much as 50% of total future adult cases can arise [7].

Childhood tuberculosis which is a marker for ongoing infection of TB in the community and indicator of level of adult TB control within the population [8, 9] poses significant threat for a resource poor country like Bangladesh for several reasons. Existing high burden of adult TB, prevalence of childhood malnutrition, poverty and high density of population in Bangladesh are among the critical ones. According to the Bangladesh National Tuberculosis Control Program (NTP), the proportion of childhood TB among all reported cases is only 3.35% [10] considerably lower compared to other high-burden countries. Contrary to Bangladesh NTP estimate, studies conducted by icddr,b and Damien Foundation Bangladesh (Rahman AS, Islam R, Ahmed T: Identification and management of tuberculosis in children in rural Bangladesh by using a simple clinical algorithm, unpublished) have shown a far greater burden (11%) of childhood TB in the community. For understanding the causal factors behind low case detection by Bangladesh NTP, the authors conducted a rapid assessment study (Islam Z, Ahmed S, Ahmed T: Rapid assessment of health system gaps in childhood TB management in Bangladesh, unpublished) in 2011 at the primary care level facilities to identify gaps in childhood TB management pertinent to capacities and resources. The assessment identified significant gaps at the selected facilities that included - lack of trained workforce, lack of information, education and communication (IEC) materials, shortage of supplies for tuberculin test reagent and radiology, poor coverage of contact tracing and Isoniazid preventive therapy (IPT).

These findings of the above-mentioned rapid assessment generated hypothesis for the study we are describing here. The hypothesis conceived that addressing gaps through tailor-made training for service providers, awareness campaign in the community and provision of adequate supplies at the primary care level can improve case detection and management of childhood TB. Accordingly, we designed this study with the objectives to improve the capacity of service providers through tailor-made training on childhood TB management, increase community awareness on child TB through targeted mass campaign and facilitate adequate and timely supply of logistics at primary care facilities.

## Methods

### Study design and sites

We conducted an implementation research with pre and post-test evaluation design from July 2012 to December 2014. Three interventions were implemented at selected primary care facilities in between cross-sectional surveys conducted in two rural sites at baseline and end line. Alongside, service statistics were collected quarterly in all three study sites. Finally, survey data and service statistics were compared to assess the change due to interventions. One urban slum area and two rural sub-districts were selected for the study in consultation with the NTP. The selection was based on higher case detection for adult TB but low for child TB. The rural sites were *Kalai* subdistrict of Joypurhat district and *Bashail* subdistrict of Tangail district having functional 50-beded health complexes. The urban site was located in Mirpur *Bawniabadh* slum area in Dhaka city. In all sites we worked closely with the NTP partner organizations namely health complex authority, BRAC in *Kalai* subdistrict and Damien Foundation Bangladesh in *Bashail* subdistrict. In urban area, we worked with the Salvation Army, Population Services and Training Centre (PSTC) and Concerned Women for Development’s (CWFD) out-patient clinics located in Mirpur *Bawniabadh* slum area.

### Study participants

The survey respondents - included selected mothers of <5 children, primary and secondary school teachers, and students, religious leaders and community leaders. They were considered as target groups for our awareness campaign. For basic and refresher training on child TB management – different categories of service providers were included e.g. doctors/managers, field workers, lab technicians, radiographers, medical assistants, village doctors and drug sellers. For facilitating supplies through periodic follow-up, we included local managers and their concerned deputies.

### The interventions

Three interventions e.g. training of service providers, mass awareness campaign and facilitation of supplies for child TB management (Table 1) were implemented in all three study areas between the baseline and end line surveys. Basic and refresher’s training sessions were organized for all graduate doctors and facilitated by child TB specialists, facility managers, pediatricians, and public health specialists. Training sessions for other service providers were facilitated by the trained investigators and field supervisors of the study team. We developed and used simplified training manuals and guidelines for doctors and field workers based on NTP’s national guidelines for child TB management.

**Table 1:** Intervention package

Intervention	Target population	Activities	Tools used	Facilitator	Place and duration
Capacity building	Graduate doctors, Fieldworkers, Lab Technicians, Radiographers, Medical Assistants Village Doctors, Drug sellers	Tailor-made training sessions on child TB management	Training manuals, Guidelines on child TB management for medical doctors, Guidelines for Field workers	Child TB specialist, Pediatrician, Investigators, Trained field supervisor	At study health complex and clinics Basic and refresher training –twice/year
Awareness campaign	Primary and secondary school students, their teachers, mothers of <5 children, community leaders, religious leaders,	Orientation, health education sessions, courtyard meetings, poster display, leaflet distribution, folksongs and street drama	Flipcharts, Posters, leaflets, Booklet on child TB in Bengali	Trained investigator and field supervisor	At classroom of schools in the study area- fortnightly, Courtyard meeting- monthly, Mosques- fortnightly, Union Council office^a^ - quarterly, Point of care at Health complex, Community Clinics, Family Welfare Centres, – weekly by rotation. Folksongs/street drama- 1–2 times per year
Facilitation of supplies	Managers of study facilities	Meetings	Checklist	Investigators Field supervisors	At study facilities- quarterly

^a^Union Council office- Lowest administrative office of the government led by an elected chairman

To increase community awareness, we focused on above-mentioned five target groups for health education/orientation sessions. Correct understanding of the target groups about the following features of child TB was considered as their knowledge: common symptoms of child TB, transmission, availability of treatment, duration of treatment, source of treatment and diagnosis, preventive measures. Necessary IEC materials e.g. flipcharts, pictorial posters, leaflets, booklets were developed in local language, displayed and distributed. Orientation sessions were regularly held at the class rooms of primary and secondary schools, out-patient –department of Health Complexes, Community Clinics, Family Welfare Centres (FWC), courtyards, mosques, *madrashas*(i.e. school of Islamic education*)* and Union Council offices (i.e. lowest administrative office of the government led by an elected chairman) located in the study areas. These sessions were facilitated by our trained study team members and assisted by fieldworkers of NTP partner organizations. In addition, we organized folk songs and street drama on child TB management inside schools and public places of the study areas twice per year.

To sensitize local managers on adequate logistic supply for child TB management- we arranged quarterly follow-up meeting at the field level and update on supplies was communicated to the appropriate authorities through them.

We collected service statistics quarterly using a checklist from all three study facilities during the study period. Finally, the baseline and end line survey data of two rural sites and service statistics of all three sites were compared to evaluate the interventions.

### Survey sample size

We assumed that 10% of the target group for survey (i.e. school teachers, students, mothers of <5 children, religious leaders, and community elites) were aware of child TB. Our intervention would increase their awareness from 10 to 40%. Sample size was calculated for each of the five selected group to detect 30% difference in the proportion of pre- and post-intervention awareness with 95% confidence level at power of 90% using design effect = 2 Using appropriate statistical formula and 10% attrition, ‘n’ stood at 92 per target group. Therefore; estimated sample size per sub-district was (92 x 5 groups) = 460 with a total sample size of (460x2) = 920.

### Sampling procedure

In each of the two sub-district lists of villages were collected from the local government office and 30 villages (i.e. clusters) were randomly selected from each list. We assumed that three to four participants of each group would be available per village (3x30 = 90 to 92) resulting in a sample size of (92 x 5 groups) = 460 in each sub-district. However; in reality we had to take more than 30 villages in some cases to get the required number of participants from different groups during baseline and end line survey. We finally interviewed 919 respondents in baseline and 915 respondents in end line survey from two sub-districts.

Earlier; lists of schools, mosques, *madrashas* and union councils were collected by the study team members and the number of teachers, students, religious leaders and community leaders – were enumerated - from where the required number of participants in each group was randomly selected. As the list of <5 mothers was not available, we followed the method of bottle spinning in a central location of a village and then moved on to one direction until we got the required number of mothers of <5 children.

### Statistical analysis

We entered data using Microsoft Access software (Office version 2007). Quality control was conducted to correct obvious errors in the data set. Questionnaire data were analyzed using IBM SPSS Statistics for Windows, version 21.0 (SPSS Inc., Chicago, IL, USA). Due to categorical nature of data, we presented our result in proportion and percentage. Sample size was calculated to detect 30% difference in the proportion for each of the selected groups with 95% confidence level and a power of 90%. We calculated change in knowledge between pre and post intervention among the survey participants using two proportions Z-test with independent groups with 5% significance level.

## Result

The school students interviewed in baseline and end line belonged to adolescent age group (i.e. older children) with an average of 14 years of age. There was no significant difference among the baseline and end line survey participants regarding their age, education and socio-economic status.

In total, 37 graduate doctors were trained in two rural sub-district and 16 were trained in the urban site during the study period. Similarly 211 health workers and 161 village doctors/drug sellers were trained in the rural sites and 38 health workers and 92 drug sellers received training in the urban site. Status of the required logistics in three field sites is given in Table 2, Stock of paediatric formulation of anti-TB drugs was limited in all study sites.

**Table 2:** Availability of logistics for child TB management

Site	X-ray	Tuberculin skin test (TST)	FNAC^1^	Sputum microscopy	Anti-TB^2^ drugs
Mirpur (urban site)	Not available	Not available	No provision	Available	Limited stock of paediatric formulation
Bashail^3^ (rural site)	Functional machine but lack of skilled radiographer	Available in limited stock with delayed replacement	No provision	Available	Limited stock of paediatric formulation
Kalai^3^ (rural Site)	Machine was non-functional in 2012–2013, later repaired by local arrangement in 2014	Available in limited stock	No provision	Available	Limited stock of paediatric formulation

^1^ *FNAC* Fine needle aspiration cytology

^2^ *TB* Tuberculosis

^3^Bashail subdistrict from Tangail district and Kalai subdistrict from Jaipurhat district

To evaluate knowledge and awareness about childhood TB in the community, a series of questions regarding common symptoms of child TB, mode of transmission, source of diagnosis and treatment etc. were asked to the participants of all five groups. The difference in proportion between baseline and end line of the respondents who could give correct answer regarding major aspects of child TB are given in Table 3. The difference in knowledge was statistically significant among all the respondent groups at the end line compared to baseline survey.

**Table 3:** Correct knowledge regarding various aspects of childhood TB

		Percentage (95% CI)^a^		
Question	Respondent	Baseline^b^	Endline^b^	*P* value^*^
Correct knowledge that TB can occur in children	Mother of <5 children	74.9 (67.1–81.3)	97.2 (93.4–98.8)	< .001
	School student	79.9 (72.1–85.9)	100%	< .001
	School teacher	87.9 (82.3–91.8)	97.3 (93.7–98.9)	< .001
	Community leader	90.5 (85.8–93.7)	97.3 (93.6–98.8)	.024
	Religious leader	86.7 (81.1–90.8)	97.8 (92.8–99.4)	< .001
Correct knowledge of symptoms of child TB (Cough > 2 weeks)	Mother of <5 children	50.38 (42.12–58.63)	90.75 (83.53–95)	< .001
	School student	62.24 (53.8–69.99)	94.59 (89.93–97.17)	< .001
	School teacher	69.18 (61.24–76.13)	94.41 (89.55–97.09)	< .001
	Community leader	55.56 (47.97–62.89)	92.66 (86.79–96.03)	< .001
	Religious leader	61.33 (53.74–68.41)	91.06 (85.88–94.46)	< .001
Correct knowledge of symptoms of child TB (Fever > 2 weeks)	Mother of <5 children	26.72 (19.53–35.39)	71.68 (62.85–79.11)	< .001
	School student	23.08 (16.65–31.06)	84.32 (77.05–89.6)	< .001
	School teacher	31.45 (23.79–40.26)	83.8 (77.11–88.81)	< .001
	Community leader	22.81 (16.84–30.13)	71.75 (63.5–78.76)	< .001
	Religious leader	24.67 (17.67–33.32)	69.83 (60.78–77.57)	< .001
Correct knowledge of symptoms of child TB (Not gaining weight)	Mother of <5 children	0.76 (0.1–5.33)	34.1 (26.04–43.2)	< .001
	School student	8.39 (4.7–14.54)	45.41 (37.19–53.88)	< .001
	School teacher	8.18 (4.77–13.66)	55.87 (49.07–62.45)	< .001
	Community leader	6.43 (3.78–10.74)	28.25 (22.28–35.09)	< .001
	Religious leader	5.33 (2.74–10.14)	37.99 (30.02–46.67)	< .001
Correct knowledge of symptoms of child TB (Reduced playfulness)	Mother of <5 children	0.76 (0.11–5.25)	25.43 (18.04–34.57)	< .001
	School student	5.59 (2.65–11.42)	34.59 (26.37–43.85)	< .001
	School teacher	8.81 (5.3–14.27)	31.28 (23.56–40.22)	< .001
	Community leader	8.19 (4.89–13.4)	28.81 (21.03–38.08)	< .001
	Religious leader	4.67 (2.09–10.07)	27.37 (19.97–36.27)	< .001
Correct knowledge of how TB is transmitted from adult to children (coughing or sneezing)	Mother of <5 children	17.05 (12.04–23.59)	68.79 (58.56–77.46)	< .001
	School student	17.48 (12–24.77)	89.67 (83.65–93.65)	< .001
	School teacher	32.9 (25.75–40.52)	86.59 (79.54–91.47)	< .001
	Community leader	18.71 (13.59–25.2)	72.88 (63.58–80.53)	< .001
	Religious leader	18.67 (13.49–25.25)	71.91 (62.3–79.86)	< .001
Correct Knowledge of duration of TB treatment (6 months)	Mother of <5 children	28.17 (18.81–39.9)	72.88 (64.62–79.82)	< .001
	School student	26.56 (17.36–38.38)	76.39 (67.73–83.29)	< .001
	School teacher	57.27 (48.32–65.78)	73.56 (64.9–80.72)	.008
	Community leader	33 (24.14–43.25)	65.22 (55.72–73.64)	< .001
	Religious leader	31.08 (21.29–42.92)	67.39 (57.81–75.71)	< .001
Correct knowledge of facility of treatment for child TB (Subdistrict Health Complex)	Mother of <5 children	59.1 (50.1–67.5)	78.1 (69.3–84.9)	< .001
	School student	52.3 (44.1–60.3)	81.6 (74.5–87.1)	< .001
	School teacher	74.4 (67.9–79.9)	86.4 (78.2–91.8)	.015
	Community leader	57.5 (48.4–66.1)	84.1 (76.6–89.5)	< .001
	Religious leader	66.1 (57.6–73.6)	81.3 (73.2–87.4)	< .001

^a^ *CI* Confidence Interval

^b^Values are presented as percentages (%) unless otherwise stated

^*^Two proportions Z-test with 5% significance level

**Fig. 1:**
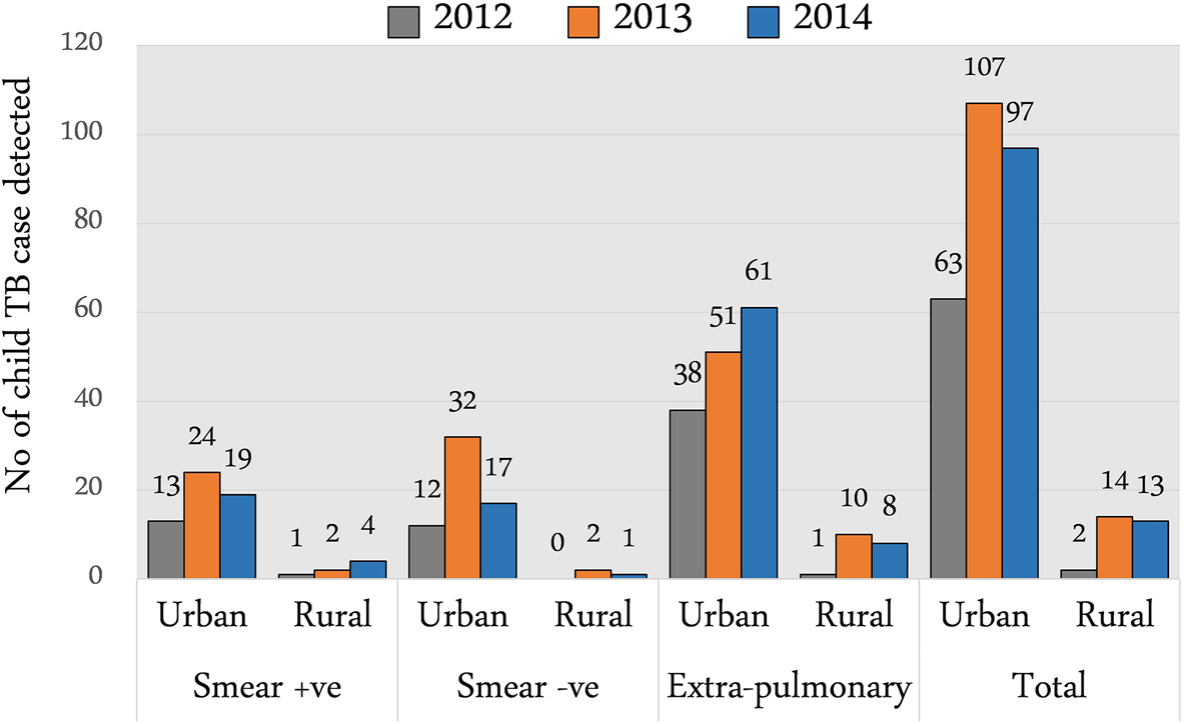
Different types of Child TB case detection across study sites

Overall, the proportion of case detection among children increased in all three sites compared to baseline with most prominent improvement was observed in the Mirpur urban slum area (Fig. 1). Prior to intervention in urban slum (baseline statistics 2012) 63 children were found registered at the study clinics suffering from TB - which increased to 107 cases in 2013 and 97 cases at the end line (2014). There was also a difference in type of child TB cases identified during the intervention period across all three sites. In rural sites at the baseline, only one patient was identified as smear positive and no smear negative case was identified. On the contrary, 13 smear positive and 12 smear negative cases were identified in 2012 in urban slum area. Number of extra-pulmonary TB cases (EPTB) was higher compared to smear positive and smear negative TB cases in all the three study sites. At the baseline, a total of 38 extra-pulmonary cases were identified in urban area compared to only one EPTB case in rural sites. The number of case detection of EPTB in children increased in subsequent years in both urban and rural sites. Fifty one cases were identified in 2013 in urban site whereas 10 cases were identified in rural sites in the same year. At the end line in 2014, 61 EPTB cases in children were identified in urban site compared to 8 cases in rural sites (Fig. 1).

**Fig. 2:**
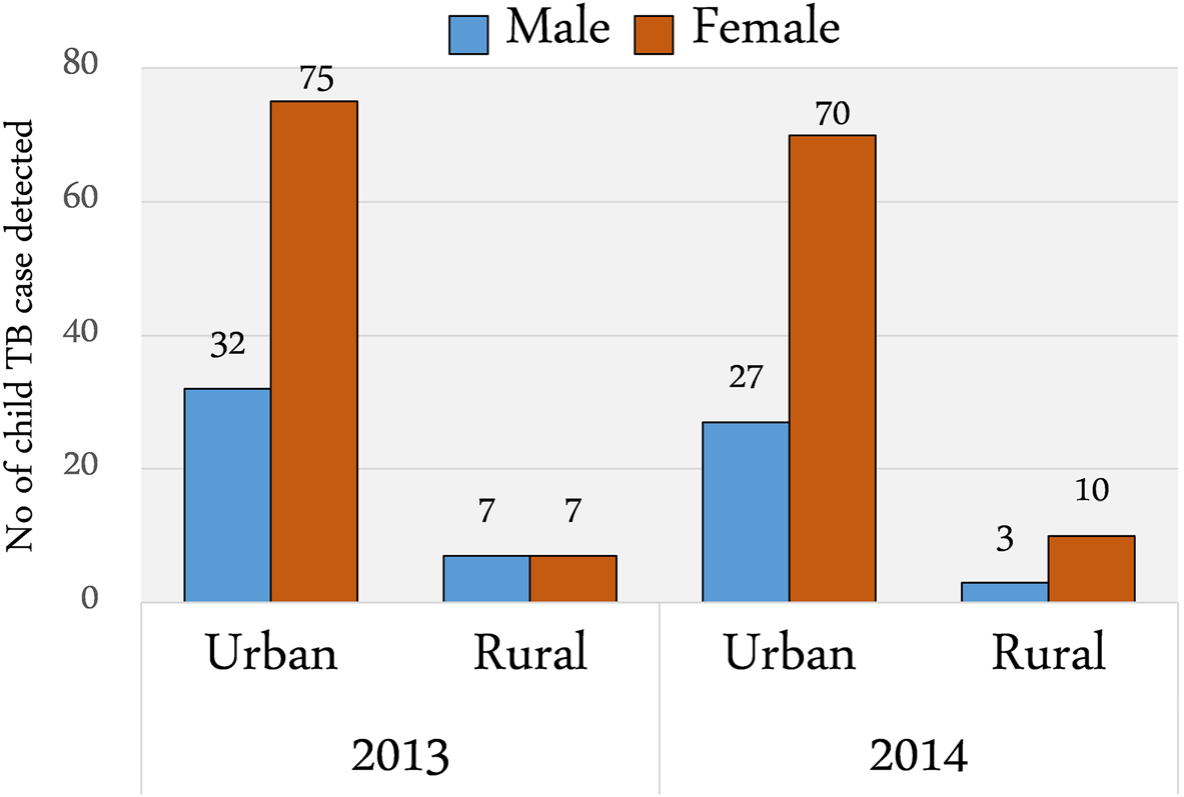
Detected Child TB cases by gender and across study sites

There was a difference in child TB cases in terms of gender more prominent in urban slum area. Out of 107 child TB cases in 2013, approximately 70% were female in urban study clinics. The proportion of male and female child TB cases was equal in rural sites in 2013 (seven male and seven female). However, 10 child TB cases (77%) were female out of 13 in 2013 in rural sites whereas 72% cases identified were female in urban site at the end line (Fig. 2). Furthermore, the majority (95%) of the child TB cases detected during 2013 and 2014 were in the age group of 10 to <15 years. Only 11 children aged <5 years were detected with TB during 2013 and 2014.

## Discussion

Through implementing the interventions we tried to create an enabling environment in the study facilities and its catchment areas which eventually resulted in improved community awareness on childhood TB with increased case detection and management during 2013 and 2014. To achieve these outcomes our interventions were aimed at reducing gaps in the service delivery systems. There was neither any IEC materials on child TB nor the service providers was adequately trained by the NTP on management of childhood TB at the time of this study back in 2012–14. Therefore; we put priority on interventions pertinent to increasing community awareness and capacity building of care givers alongside facilitation of supplies at the primary care level facilities. It is notable that the study team members were not directly involved in case detection or active screening but facilitated training and mass orientation sessions in collaboration with the partner organizations.

The total number of child TB cases detected in 2013 and 2014 in Bawniabadh, Mirpur urban site was relatively high compared to other two rural sites and the trend remained higher with extra-pulmonary cases throughout the intervention period. This particular slum area is highly crowded and transmission of Mycobacterium Tuberculosis is strongly associated with degree of crowding [9, 11]. This higher case detection in urban site could be linked to the fact that a large proportion of its catchment population in *Bawniabadh* slums had better access to tertiary level TB hospitals and other hospitals in Dhaka city. Suspects of this area might have availed the opportunity of getting their diagnosis made at those well-equipped hospitals and showed up at the study clinics for DOTs in most of the cases. Our two rural sites were relatively less crowded and far away from such tertiary facility. Furthermore; the health workers of partner non-governmental organizations (NGO) engaged in urban locations were more vigilant compared to government health workers of the other two rural sites. Similar findings were observed in Malawi, where there was a difference in incidence rate between urban and rural areas supposedly due to under-diagnosis at health centres or poor access to medical facilities for rural people [12]. Differences in TB incidence between urban and rural areas have been demonstrated in other countries also [13]. Certain urban groups like slum dwellers remain as high risk population due to exposure to adverse environmental conditions (overcrowding, poor living conditions) [14]. S banu et al. in their study in the similar setting from Dhaka Bangladesh found two times higher prevalence of TB in urban setting compared to national data attributed to overcrowding [15].

To establish an accurate diagnosis for childhood TB is a critical obstacle as less than 15% of cases are sputum acid-fast bacilli smear positive, and mycobacterial culture yields are 30%–40% [16–18]. In our study we found overall proportion of EPTB was 56% (130 children) and lung TB cases was 44% (101 children) in 2013, 2014. Confirmation of lung TB cases took place at the study facility level while EPTB cases were confirmed by secondary/tertiary level hospitals/private diagnostic centers - as fine needle aspiration cytology (FNAC) test was not available in study facilities. However; all cases received treatment at the study facilities. Higher number of extra-pulmonary TB in children warrants immediate attention of the program to address the need of FNAC test at the primary care level or an effective facilitated referral system for that. Only recently it has been speculated that under-detection of child TB in the country is due to an almost exclusive focus on ‘sputum smear-positive TB’ and neglecting the true burden of EPTB in children [10]. Recognizing the shortfall, Bangladesh NTP is currently emphasizing on training of health professionals on child TB management across the country.

Surprisingly in this study, the number of female cases detected was higher compared to male cases and this happened irrespective of urban and rural study sites. This finding opposes the usual knowledge and findings from previous studies done in adult population. Studies carried in Bangladesh observed gender difference in routine tuberculosis diagnosis where women were as much as 3 times less likely to be diagnosed [19, 20]. However, our finding was also comparable with the author’s earlier study (Rahman AS, Islam R, Ahmed T: Identification and management of tuberculosis in children in rural Bangladesh by using a simple clinical algorithm, unpublished) in *Madhupur-Dhanbari* of Tangail district and NTP’s service statistics. Plausible explanation of such gender difference in child TB cases is not yet available.

It is evident from comparison of baseline and end line survey data that community awareness among target groups increased significantly –which even exceeded our estimated limit of 30% between pre and post-test evaluation. The long existing gap in IEC materials, training of service providers and community orientation was adequately addressed by the study team through developing and distributing posters, leaflets, handbooks, folksongs and flipcharts on childhood TB management. It has proven instrumental in raising mass awareness as well as capacity of service providers. As mentioned earlier, it should be noted that at the time of this study neither NTP nor any other NGO, print or electronic media had any IEC program focusing exclusively on child TB. So this is reasonable to argue the improvement of knowledge among the target groups regarding child TB can be attributed to our awareness building intervention. Our finding on awareness campaign indicates that program uptake of these IEC materials could be very useful for Bangladesh NTP in raising community awareness on childhood TB. Evidence from other high burden countries shows that implementation of awareness campaign as well as involving general practitioners increased case finding and preventive therapy [21]. It has been stated by WHO that childhood TB can only be addressed effectively through collaboration across the health system and community. Community leaders and community based organizations can be the key stakeholders in the community and act as actors to promote child TB education and awareness [21]. The Bangladesh NTP and its partners have been engaged in Advocacy, Communication and Social Mobilization (ACSM) campaigns, principally funded by the Global Fund to fight AIDS, TB and Malaria (GFATM). These campaigns are exclusively aimed at increasing mass awareness about TB in adult [22]. However, a paper has recently been published on the knowledge and associated factors regarding adult TB based on a nationwide survey in Bangladesh [23]. This study found that approximately 67% of respondents knew the mode of TB transmission in adults. Being a female had 2 times higher odds of having poor knowledge about adult TB. Another study [24] done among TB patients at urban DOTS centres in Bangladesh documented even poorer knowledge about adult TB where only 56% knew accurate mode of TB transmission. This proves lack of awareness in the community even for adult TB.

We observed chronic shortage of Tuberculin skin test (TST), problem with functional radiology and no provision of FNAC at both the rural study sites and no provision of x-ray and FNAC at urban study clinics. Paediatric formulation of anti-tubercular drugs was often replaced by adult formulation in broken form/divided doses. The availability of essential logistics to manage childhood TB at the primary care level did not improve adequately despite quarterly meetings with the respective authorities. This remains as one of the key areas of further research in strengthening child TB management in Bangladesh.

### Limitation of the study

Transfer and or deputation of government doctors from the study health complexes to other places during the intervention have affected the expected outcomes of our study. Change in awareness on child TB was measured in the two rural sites and not in the urban site due to time and budgetary limitations. Furthermore, there was a nationwide political unrest during the last quarter of 2013 and movement was seriously affected for general people which might have affected case detection particularly in two rural sites.

## Conclusion

Despite having constraints in diagnostic supplies at the study facilities, transfer of trained government doctors and political unrest during the study period - the proportion of childhood TB cases among all reported TB cases increased in all 3 study facilities that had exceeded the NTP estimate of 3%. Following mass campaign; community awareness on childhood TB increased significantly across all study sites that exceeded our anticipation. The National TB Control Program should immediately introduce IEC materials on childhood TB (i.e. poster, leaflet, flipchart etc.) alongside increasing capacity of service providers, availability of diagnostics (i.e. X-ray, tuberculin skin test) and paediatric formulation of anti-tubercular drugs at the primary care level.

## Abbreviations

ACSM: Advocacy, communication and social mobilization campaigns
CWFD: Concerned women for development
EPTB: Extra-pulmonary TB
FNAC: Fine needle aspiration cytology
GFATM: The global fund to fight aids, tuberculosis and malaria
icddr,b: International centre for diarrhoeal disease research, Bangladesh
IEC: Information, education and communication
IPT: Isoniazid preventive therapy
NGO: Non-governmental organization
PSTC: Population services and training centre (PSTC)
TB: Tuberculosis
TST: Tuberculin skin test
WHO: World health organization
